# Quantifying forensically relevant vertebrate scavenging: a pilot study to develop a methodological framework using Cape grey mongoose (*Galerella pulverulenta*) as an illustrative model

**DOI:** 10.1093/fsr/owae069

**Published:** 2024-12-03

**Authors:** Devin Alexander Finaughty, Gabriella May French, Kara Sierra Adams, Maximilian Jan Spies, Victoria Elaine Gibbon

**Affiliations:** Human Variation and Identification Research Unit (HVIRU), Department of Anatomical Sciences, School of Biomedical Sciences, Faculty of Health Sciences, University of the Witwatersrand, Johannesburg, South Africa; Division of Clinical Anatomy & Biological Anthropology, Department of Human Biology, Faculty of Health Sciences, University of Cape Town, Cape Town, South Africa; School of Anthropology and Conservation, Division of Human and Social Sciences, University of Kent, UK; Division of Clinical Anatomy & Biological Anthropology, Department of Human Biology, Faculty of Health Sciences, University of Cape Town, Cape Town, South Africa; Division of Clinical Anatomy & Biological Anthropology, Department of Human Biology, Faculty of Health Sciences, University of Cape Town, Cape Town, South Africa; Division of Clinical Anatomy & Biological Anthropology, Department of Human Biology, Faculty of Health Sciences, University of Cape Town, Cape Town, South Africa

**Keywords:** forensic anthropology, forensic taphonomy, Cape Town, postmortem interval, decomposition, scavenging, Cape grey mongoose

## Abstract

Currently, forensic death investigations in the Western Cape, South Africa do not account for vertebrate scavenging activity; however, previous research in the city of Cape Town has shown a significant impact on the rate of decomposition due to scavenging by the local Cape grey mongoose (*Galerella pulverulenta*). This pilot study aimed to develop a framework to more robustly quantifiably describe and analyse the scavenging behaviour of this species on a single clothed 60 kg porcine carcass in a forensically significant location in Cape Town (i.e., a region of the city with a proportionately large forensic case load). Feeding behaviours are the focus of this framework and analysis, but non-feeding behaviours in the immediate vicinity of the carcass are also distinguished and described. Additionally, it was assessed whether all behaviours varied in their frequency of occurrence as decomposition progressed. More than 40 h of mongoose interaction with the carcass were recorded using motion-activated infrared-capable wildlife trail camera videography. The highest frequencies of scavenging activity were observed in the abdominal and the eye socket regions of the carcass. Abdominal feeding largely involved soft tissue modification, whereas more intensive activity in the eye socket suggested a higher likelihood of hard tissue scavenger artefacts being found there. A Kruskal-Wallis test confirmed that some feeding behaviours occur more frequently during specific decomposition stages. For example, scratching is common during the earlier stages of decomposition, ostensibly to create an opening in the flesh. Twisting whilst biting was only observed in latter stages of decomposition, likely due to the increased toughness of the soft tissue as it desiccated. This pilot research offers detailed insight into scavenger behaviours previously unreported in the forensic taphonomic literature, and proposes a provisional method of quantifiable analyses of scavenger behaviours that extends what has previously been published in the forensic taphonomic literature. Local validation of the observations is planned, and international replication of the research for diverse scavenger guilds is encouraged.

**Key points:**

- Novel framework for detailed quantitative analysis of vertebrate scavenger behavior using the Cape grey mongoose as a model.
- First systematic study of scavenger behaviour in South Africa and likely Africa-wide.
- Cape grey mongoose preying on blow fly larvae, emphasizing the necessity of videographic documentation.

## Introduction

Forensic taphonomy is a transdisciplinary subject concerned with events that occur following an individual’s death until the point of recovery of their remains [1]. Forensic death investigations frequently seek to ascertain time-since-death (otherwise known as the postmortem interval or PMI) and cause-of-death. Achieving these goals requires knowledge of postmortem processes in the environment from which the remains were recovered, along with the ability to delineate those that have occurred ante- and perimortem [2]. Local knowledge is key, as the rate and process of decomposition can be drastically altered by many environmental factors, one being the consumption of remains by vertebrate and invertebrate scavengers [3, 4]. Carrion is a critical food source for many vertebrate species across the animal kingdom which facultatively scavenge when carrion is available, including non-carnivorous species [5–7]. Internationally, numerous species of potential forensic significance have been identified. These include domestic or feral cats and dogs, wolves, coyotes, foxes, badgers, bobcats, bears, wild pigs, avian species like vultures and corvids, as well as smaller vertebrates like racoons, opossums, skunks, fishers, mongooses, and various rodents [8–34].

Currently, PMI methodologies in forensic death investigations do not quantitatively account for vertebrate scavenging activity [35]. The substantial impact on baseline decomposition rate has been previously described ([36] and articles cited above); therefore, its inclusion in forensic taphonomic studies is vital for the data to be forensically realistic and accurate. In some circumstances (e.g., regions where scavengers are prevalent and/or their usual food sources are scarce or more energetically expensive to obtain compared to scavenging), certain vertebrate species can play a prominent role in the rapid breakdown of remains [25, 37]. Scavengers may also scatter remains when assemblages become disarticulated; another critical reason for identifying local scavengers [36].

Ubelaker [38] draws attention to the role of scavenging during decomposition and highlights distinctive species-specific patterns that may be left by those animals using the carrion resource. For example, canids will often remove the extremities from carrion before moving onto the mid-section [8]. Scavengers may also leave identifiable artefacts (tissue-based markers/evidence) on bones and/or soft-tissue, such as punctures, scrape marks, and shallow scratch marks. These can be misinterpreted as peri- or antemortem trauma, so a thorough understanding of the local scavengers and their common behaviour in each environment is essential for taphonomic research [8, 39]. Given the widespread scavenging by vertebrates in habitats around the world, identifying whether scavenging has occurred, and by which animal, can provide crucial information regarding the PMI, the context of the remains, and where they may be scattered. Herein lies the fundamental motivation for the current investigation.

In a local context, previous research by Finaughty [40] in Delft, Cape Town identified the Cape grey mongoose (*G. pulverulenta*) as a prolific diurnal scavenger in the forensically relevant Cape Flats Dune Strandveld habitat—capable of altering the decomposition rate of human adult-sized porcine carcasses. These observations stood in contrast to prior published literature on this species [41, 42] where their diet was documented to comprise >90% small vertebrates. Subsequent research in the same region [37, 43–46] has supported these initial observations regarding diet, along with other previously published aspects of this species’ biology (i.e., that they are strictly diurnal [47, 48]; favour dense vegetation for foraging [41, 42, 49]; and partake in inter-individual sociality where food is concerned when they are an otherwise solitary species [49]). Although the local research has documented the patterns of feeding and scattering of domestic pig carcasses for the Cape grey mongoose, along with similar behaviour recorded during a forensic death investigation from a comparable species (the yellow mongoose, *Cynictis penicillata*) [43], no clearly discernible artefacts of scavenging or feeding behaviours for the Cape grey mongoose have been previously identified or described. Such artefacts would assist in confirming the presence of scavenging in the first instance and allow for the subsequent alteration in decay rate to be factored into PMI estimation during forensic death investigations.

Understanding the impact that mongoose scavenging can have on the decomposition process and preservation of a set of remains requires the identification of a distinct artefact, or suite of artefacts, that indicates scavenging has occurred in the first place. Unlike larger canid and ursid scavengers who leave distinct impressions upon hard tissues [8, 9], mongooses—with their small carnassial dentition that is not geared towards large animal hard tissue exploitation—may not leave such obvious artefacts on forensically relevant human remains (or equivalently sized animal remains). As a result, their (potentially considerable) impact on the rate and pattern of decomposition may not be accounted for during forensic taphonomic and anthropological appraisals. This pilot study, therefore, builds upon previous work analysing and describing Cape grey mongoose feeding behaviour in a forensically significant location in Cape Town, South Africa, as well as international efforts to describe vertebrate scavenger behaviour. We have four aims: (1) to develop a methodological framework for classifying scavenging behaviours such that they may be quantifiably analysed at the individual behaviour (rather than global generic “scavenging behaviour”) level; (2) to determine whether it is possible to identify regions on a carcass of preference to mongooses; (3) to determine whether specific feeding behaviours can be observed and/or associated with these carcass regions or tissue types; and (4) to determine whether any of the identified behaviours may leave lasting artefacts useful for identification of scavenging. We are interested to learn whether mongoose feeding behaviour varies during the stages of the decomposition cycle; if, during feeding, scavenging frequency varies depending on the carcass region; whether specific feeding behaviours are utilized by the mongoose for different body regions; whether the mongooses feed on Dipteran larvae (maggots) and, if yes, whether this preferred over feeding on the carcass and how might it be influenced by maggot mass size.

## Materials and methods

**Figure 1:**
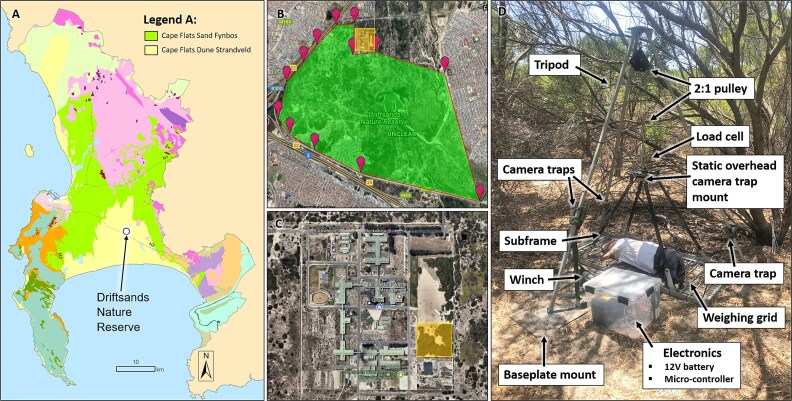
(A)The approximate location of the research site on the Cape peninsula at the most southwestern tip of South Africa (modified map from Wikipedia Commons). (B) Driftsands Nature Reserve is highlighted in the image as a large, irregular shape, and the research facility's location relative to Driftsands is highlighted as a small rectangle near the top-center of the image (satellite image sourced from Google Maps). (C) The specific location within the research facility where the research was conducted is highlighted with a small square towards the bottom right of the image (satellite image sourced from Google Maps). (D) The experimental setup showcasing the automated carcass weighing apparatus.

This research was conducted at a secure research facility located in the suburb of Delft, Cape Town, South Africa (Figure 1A–C). Delft lies in the heavily populated area of the city known as the Cape Flats—a sweep of low-land located between two mountainous areas: Table Mountain to the west and the Boland and Hottentots-Holland mountains to the east [40, 50]. The facility is surrounded by a 6 ft (1.8 m) electric security fence but is generally porous to small and medium-size animals who can dig through the soft sand beneath it or slip through the gaps in the wire strands (~20 cm apart), hence the unincumbered presence of Cape grey mongoose on the site. Other animals noted to frequent the site include domestic cats (*Felis catus*), a variety of small rodents (e.g., brown rats (*Rattus norvegicus*), Cape dune mole-rats (*Bathyergus suillus*)), corvids (e.g., Cape crow (*Corvus capensis*), pied crow (*Corvus albus*), white-necked raven (*Corvus albicollis*)), and some raptors (e.g., Cape eagle owl (*Bubo capensis*)).

A single 60 kg clothed porcine body (*Sus scrofa domesticus)* was utilized as the carrion source. The pig was terminated *via* a single 0.22 LR calibre gunshot delivered to the brain, with death confirmed by a State veterinarian, clothed, and deployed during the morning of January 13th, 2020. Ethical approval for porcine carcass use and the termination procedure was granted by the University of Cape Town’s Faculty of Health Science’s Animal Ethics Committee (UCT FHS AEC Ref. No.: 018_023). The projectile entrance wound created by the termination protocol was small (<5 mm in diameter) and rapidly clotted with blood, with no exit wound (i.e., the projectile remains lodged within the cranial cavity). Previous research in this region using porcine bodies terminated *via* the same methodology, conducted over multiple years and across variable seasons [37, 40, 43, 51] has not found the wound to be a cause of considerable deviation in the decomposition process (e.g., it does not attract blow fly oviposition). A clothed porcine body was used as it formed part of a larger research project investigating the effect of clothing on decomposition and scavenging behaviour in the region [44, 45]. The body was photographed from a static, standardized overhead position; the resulting photographs permitted measurements of the body to be captured using the software ImageJ (https://imagej.net/ij/). The body was placed on a weighing grid with known grid size squares of 10 × 10 cm, underlaid with bird wire with gap dimensions of 2.5 × 2.5 cm. The large gaps within the grid ensured good body-soil contact, whilst the thin bird wire prevented the loss of any sizeable elements that might notably alter accurate measurement of the body weight. This enabled the calibration of the digital measuring tool. Daily body weight loss was employed as a quantitative measure of the rate of decomposition [52, 53]. The first weight of the body was recorded by the farmer following the termination protocol. An automated body weighing apparatus (Figure 1D) was deployed [51, 54, 55], wherein the sub-frame and weighing grid bearing the porcine body, stabilized by a tripod, is lifted and weighed once at night to reduce disruptions to the scavenging guild [41, 42, 49, 56]. Four infrared-capable motion-activated wildlife trail cameras (camera traps), recording 60 s of video footage per activation with a rearming interval of 10 s, were suspended from the legs of the weighing apparatus tripod between 30 and 70 cm away from the body, angled to view the body’s abdomen, rear, head, and a close-up of the head. They were positioned to avoid body obstruction while simultaneously permitting clear observation of scavenging behaviour. The overhead camera trap was a Foxelli Oak’s Eye 2, and the three surrounding camera traps included two Primos Proof Camera 03’s and one Bushnell Trophy Cam 119 436.

Data were collected for 70 days, with data collection ceasing on March 23, 2020, once the porcine body had reached the following criteria:

- Skeletonization was reached (any one of the following three) [57];
  - Obvious loss of internal abdominal structure, only spine and ribs remaining underneath dried skin;
  - Substantial unweathered/greasy bone exposed (>50% of the body) and no wet decomposition when observed underneath the body (facilitated by the grid with block and tackle);
  - Significant areas (>30% of the body) of bleached or weathered bone exposed.
- The weekly accumulated weight loss declined to below 5% of the original weight value for three consecutive weeks;
- Minimal insect appearance/activity.

The camera trap data were analysed, and the stages of decomposition recorded according to classification by Goff [58], into four broad stages: early decomposition, indicated by discolouration of the remains, the presence of fly activity, and the onset of bloating; active decomposition, distinguished by the deflation of the decomposing body following bloating, with large maggot masses inhabiting the decomposing body; advanced decomposition, identified by the lack of insect infestation and a noticeable, visual decrease in body mass; and dry remains, categorized by little to no remaining soft tissue or completely skeletonized remains. To augment qualitative descriptions of decomposition progression, daily weight measurements were used as an indication of the decomposition stage; the higher the weight, the earlier the porcine body was in the decomposition process.

The analyses of scavenging behaviour were conducted using Timelapse (open-source software; [59]). Data were screened and cleaned to remove null triggers (those that did not feature any potential/active scavengers) and corrupted files, leaving only data that included the visitation of mongooses (and any other potential scavengers that might leave artefacts to account for artefacts that might not be left by mongooses specifically). Recurrent behaviours were identified and defined, whereafter frequencies of each behaviour and body regions where feeding behaviours were exercised were recorded. Only feeding behaviours that exclusively related to the Cape grey mongoose were diagnosed and defined, given that this was the only vertebrate documented to scavenge the carcass.

**Figure 2:**
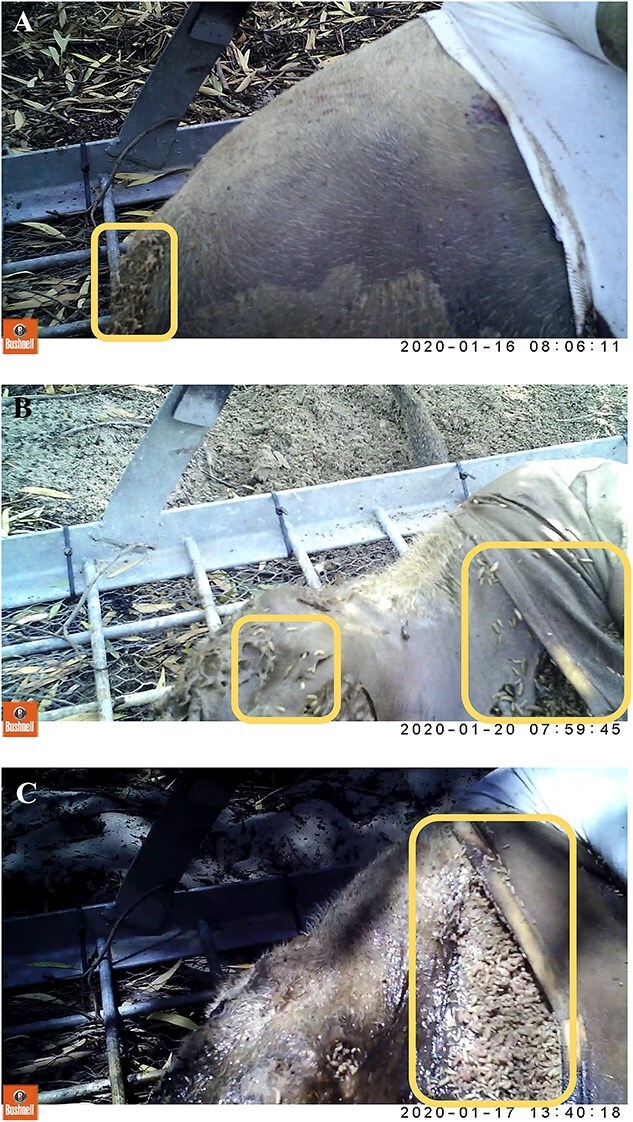
Illustration of “small” (A), “medium” (B) and “large” (C) maggot mass per definitions (mass circled), respectively.

Maggot mass sizes are ever-changing during the decomposition process, leaving the distinction between general size descriptions susceptible to subjectivity. During this study, visuals of maggot masses were oftentimes blocked or obscured due to the presence of clothing on the carcass, meaning that digital measurements of the masses could not be taken. To estimate how the mass sizes changed over time, in relation to the overall size of the porcine carcass, definitions were created using three general groups; “small”, “medium”, and “large”. Figures 2A–C were all taken from the motion-activated camera situated closest to the body’s head, and illustrate the differences between these groups when compared against the same body region.

- Small maggot mass (Figure 2A): a cluster of maggots covering less than a quarter of the observed surface area of the body, with little to no dispersion of the mass. Movement of the mass is noticeable, but remains contained to identified area.
- Medium maggot mass (Figure 2B): a cluster of maggots that may cover up to half of the observed surface area of the body, with some dispersion of the mass occurring. Movement is noticeable at the origin point, where the bulk of the cluster remains, and may be visible beneath the epidermis.
- Large maggot mass (Figure 2C): a cluster of maggots covering at least half of the observed surface area of the body, with some dispersion of the mass occurring. Movement of the mass is pronounced and evidence of below-epidermis infestation is present.

The variable pairs that were tested for relationship significance were as follows: porcine body region and the number of mongooses; daily weight and decomposition stage; and body region and decomposition state. Pairwise comparisons were completed using a Kruskal-Wallis (K-W) test with a Bonferroni correction formula for multiple comparisons that compared scores between feeding behaviours that were identified and daily weight measurements. Feeding behaviours were categorized into three groups for two reasons: the first is to ensure they met the requirements for a K-W test (i.e., the test must include one continuous, dependent variable—daily weight—that can be tested against a categorical, independent variable with three or more categories—feeding behaviours). The second reason was that specific feeding behaviours were frequently documented to be utilized together, and the groupings were formed based on the frequency of behaviours being utilized together. For example, biting and tearing were seen more frequently together than biting and burrowing snout. Spearman’s $ \rho $ tests were used to indicate whether the number of mongooses scavenging and decomposition state had an impact on the extent of scavenging by body region. Pairwise comparisons were conducted using Dunn’s (1964) procedure with a Bonferroni correction formula for multiple comparisons.

## Results and discussion

A total of 1 650 video segments were analysed for evidence of scavenging. The Cape grey mongoose was the only vertebrate documented to scavenge the remains. Table 1 records the total scavenging times and decomposition stages. In total, mongooses interacted with the body for 45 h 20 min and 30 s. Much of this time saw the mongooses actively scavenging the remains, but an exact timeframe was not ascertained as the feeding behaviours did not always occur for the entire 60 s of a recording and, in some cases, multiple mongooses interacted with the decomposing body simultaneously. The number of days the body spent in each decomposition stage was also recorded (Table 1). As previously observed, mongoose activity was limited to daylight hours, further confirming their diurnal rhythms [37, 40, 42–44, 49, 56].

**Table 1:** The total time the Cape grey mongoose was observed scavenging the carcass split by both the decomposition state and the number of mongooses scavenging.

Decomposition state	Duration (days)	Total time: all scavenging (hh:mm:ss)	Total time: single mongoose visiting (hh:mm:ss)	Total time: multiple mongooses visiting (hh:mm:ss)	Total number of visits
Early	4	20:27:38	19:08:02	01:19:36	104
Active	5	21:30:52	20:32:28	00:58:24	90
Advanced	61	03:22:00	02:59:16	00:22:44	690
Dry	0	00:00:00	00:00:00	00:00:00	0
Total	70	45:20:30	42:39:46	02:40:44	884

In 1 360 video segments, 1 517 distinguishable scavenging instances were identified. The following feeding-specific behaviours were observed and are defined in Table 2: licking (*n* = 93, 6%), scratching (*n* = 47, 3%), burrowing snout (*n* = 120, 8%), biting (*n* = 509, 34%), pulling (*n* = 497, 33%), tearing (*n* = 126, 8%), chewing (*n* = 100, 7%), gnawing (*n* = 21, 1%), and twist-with-bite (*n* = 4, 0.3%). These behaviours were examined across body regions and by decomposition stage, as detailed in the sections below.

**Table 2:** The list of feeding characteristics and definitions identified during the study.

Behaviour	Definition
Licking	The tongue is repeatedly run over an area on the carcass.
Scratching	The use of either one or both paws, extending the claws, to create an opening in the flesh. The use of both paws resembling a digging manoeuvre. The use of one paw to steady their weight whilst continually running the other paw over the same part with claws extended.
Burrowing snout	The snout is pushed into a feeding location, often a cavity, and with a combination of biting and licking to reach the resource.
Biting	Part of the carcass is held between the maxilla and mandible.
Pulling	Whilst biting, the scavenger pulls their head and body away from the carcass using the front paws for stabilization, attempting to break off part of the carcass.
Tearing	Often done in tandem with biting and pulling; the head is turned to the side whilst holding soft tissue in the mouth. This allows less flexibility of the flesh, increasing the success of breaking off pieces of the carcass.
Chewing	A succession of small bites in the same place with the molars, loosening small parts of carcass flesh, whilst the head is facing slightly left or right.
Gnawing	Whilst holding part of the carcass in the maxillary and mandibular incisors, small biting motions are made.
Twist-with-bite	Whilst biting the carcass, the head is turned ~180° in quick succession, attempting to dislodge a piece of soft tissue from the carcass.

### Scavenging activity by decomposing body regions

In 642 video segments, the specific region on the porcine body where feeding by Cape grey mongoose took place was determined. A cross-tabulated chart recording the frequencies of the different feeding behaviours as they occurred at each body region is presented in Table 3. Overall, no significant differences were observed between the number of mongooses and the extent of scavenging by the body region ($ \rho $ = 0.111, *n* = 478, *P* = 0.15).

**Table 3:** Recorded frequencies of feeding behaviours as they occurred on each region of the carcass. *“Total N”* refers to the number of times each feeding behaviour has been recorded within a valid video segment. A single video segment could showcase a behaviour occurring at multiple regions of the carcass and/or multiple behaviours occurring simultaneously.

	Total	Head	Eye Socket	Ears	Neck	Arms	Legs	Abdomen	Back	Rear
Licking	93	26	37	6	7	0	0	15	2	0
Scratching	47	5	1	3	10	0	0	20	7	1
Burrowing snout	120	21	87	8	1	0	0	3	0	0
Biting	509	142	55	61	77	1	3	161	7	2
Pulling	497	140	54	60	76	0	3	155	7	2
Tearing	126	43	1	10	25	0	2	45	0	0
Chewing	100	14	12	17	9	1	3	42	2	0
Gnawing	21	2	2	2	4	1	2	8	0	0
Twist-with-bite	4	0	0	0	2	0	0	2	0	0
Total	1 517	393	249	167	211	3	13	451	25	5

The total time mongooses spent scavenging at a particular region of the body was a poor predictor of the likelihood of identifying scavenging artefacts in that region. Rather, specific feeding behaviours used by the mongooses to consume tissue in each region of the body proved to be a better indicator of the likelihood of finding artefacts associated with those feeding behaviours. To illustrate, some feeding behaviours, such as licking, were recorded frequently (*n* = 93, 6%), but left inconspicuous marks on the body that are unlikely to withstand the decomposition process. Similarly, if we consider the frequency of scavenging at the abdomen alone (*n* = 451, 30%), the abdomen may appear to be the most likely site to present identifying markers of Cape grey mongoose scavenging. However, when tissue type is considered, the validity of this conclusion decreases. The tissue type “skin” was only marked once for scavenging at the abdomen, whereas “decomposed flesh” was frequently fed upon in this body region compared to other recorded instances of scavenging “decomposed flesh”. Over time, the skin, unless mummified, will decompose to the point of nonexistence [58]. Carrying this through to forensic cases, markers left by the Cape grey mongoose on the skin are only relevant if the remains are recovered before the next phase of decomposition begins and/or the remains mummify. It is worth noting that camera trap failure during the first week of deployment may have resulted in fewer recorded instances of skin marking, but the conclusion still stands.

Turning attention to the subcutaneous tissue, “biting” and “pulling” behaviours were observed most on the abdomen and head; moderately on the neck, ears, and eye socket; and least on the back, rear, and limbs. These feeding behaviours were the most frequently observed in general, each with instance counts over 400. Two areas experienced high volumes of scavenger activity—the eye socket (*n* = 249, 16%) and ears (*n* = 167, 11%). Feeding at the eye socket largely revolved around removing the vitreous contents during the early days of the deployment. However, Cape grey mongooses continued to return to this area even after vitreous matter could no longer be visually identified. The “burrowing snout” behaviour was the most commonly employed behaviour in this body region (35% of total behaviours recorded for the eye socket), closely followed by the related consumptive behaviours of “biting” (22%) and “pulling” (22%). Once the snout was inserted, the mongooses would move their mandible in a manner similar to “chewing” or “gnawing”. The eye socket is a relatively small cavity, making it probable that the mongoose snouts were pressed into this cavity to scrape any remaining vitreous contents from the borders of the orbital foramen with their incisors, providing a distinct possibility for scavenging artefacts to be present in the region of the body. Moreover, this behaviour suggests their feeding strategy and the associated behaviours are adapted to the anatomical morphology of the target region.

Decomposing body consumption sequences relating to preferred areas on a body are extremely under-researched, and more so when the animal in question has not been recognised as a carrion scavenger until recently [37, 40, 44]. Recent research has noted a common theme of the Cape grey mongoose utilising the anus and groin as access points for body tissue [37, 40, 44]. These observations deviate from those presented in this study, as no scavenging activities were documented in these areas on the body. There are several plausible explanations for this: the most likely is that the clothing (specifically, the denim jeans secured with a belt) posed an access barrier to the anal region, driving the mongooses to the abdomen. The state of dress of the deceased should, therefore, be considered when evaluating the body to identify potential regions bearing scavenger artefacts.

The deviated feeding pattern may also be due to the decreased number of decomposing bodies available at the site. In Spies’ previous studies [37, 43], three porcine bodies were deployed simultaneously. Currently, all research at the site has found mongoose scavenging to be most abundant in the earlier stages of decomposition [37, 40, 44]. If numerous bodies are deployed at once, the Cape grey mongoose may be inclined towards using existing orifices for edible body tissue access, rather than utilising additional energy to create a new entry point. Thus, capitalising on the abundance of the ephemeral resources available with minimal energy expenditure. Only one body was deployed for this study. This resource reduction may drive the Cape grey mongoose to implement a “waste not, want not” approach; they are liable to engage in the costly process of creating a new entry point, e.g., in the abdomen, as the single porcine body was the only resource available.

### Scavenging behaviours through the decomposition process

Scavenging behaviours by the Cape grey mongoose across the decomposition cycle were most frequent during the early and active stages of decomposition, with simultaneous scavenging by multiple mongooses occurring more frequently during early decomposition and single mongoose visits occurring more during active decomposition. For both single and multi-mongoose visits, there was a noticeable drop-off in scavenging activity at the onset of advanced decomposition. That said, when the decomposition state and scavenging observed were analysed by porcine body region, no significant differences were found ($ \rho $ = 0.068, *n* = 4 318, *P* = 0.139). However, when examining specific behaviours significant differences were observed, as explained below.

**Figure 3:**
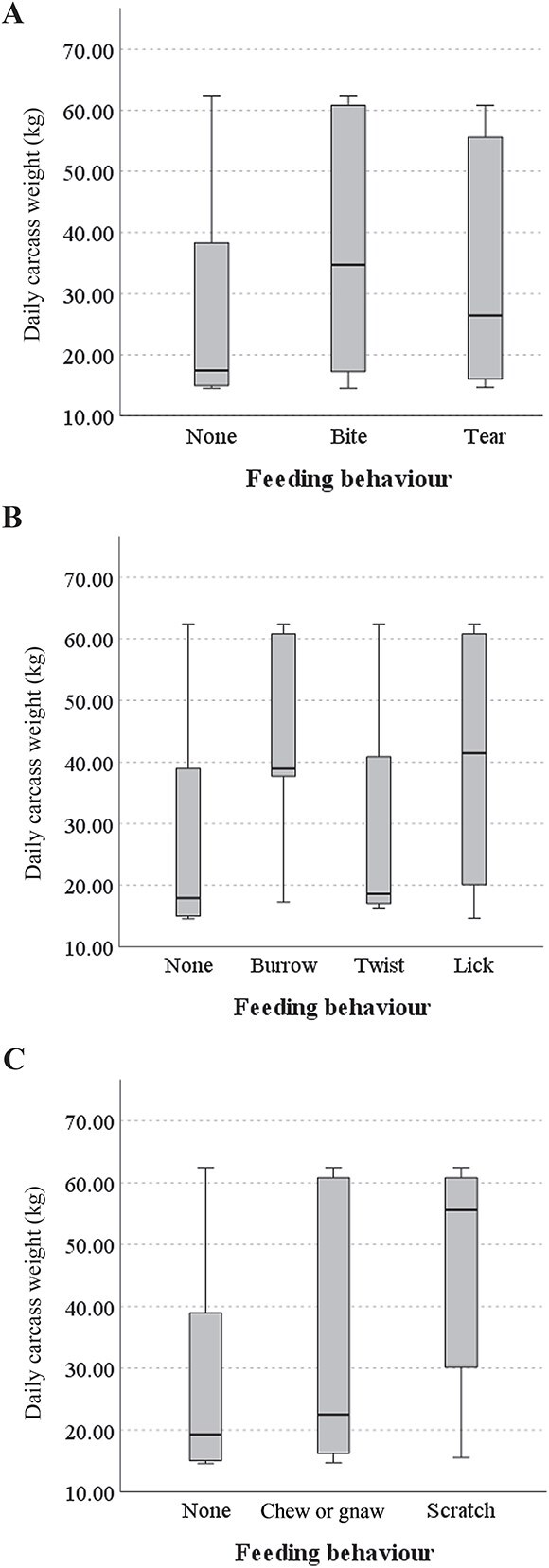
(A) Distribution of “bite” and “tear” behaviours relative to carcass weight. (B) Distribution of “burrow”, “twist”, and “lick” behaviours relative to carcass weight. (C) Distribution of “chew or gnaw” and “scratch” behaviours relative to carcass weight.

The first group of behaviours (H = 70.506) included “none” (no behaviours observed) (*n* = 3 855, M = 17.43 kg), “biting” (*n* = 347, M = 34.73 kg), and “tearing” (*n* = 116, M = 26.42 kg). *Post-hoc* analyses showed statistical significance in median feeding behaviour scores between “none” and “bite” behaviour (*P* = 0.000), but not between the “tearing” feeding behaviour and any other combination. Thus, the null hypothesis that the distribution of daily weight would be the same across all feeding behaviour categories was rejected. Figure 3A depicts the distributions observed in this test. From these box plots, it was apparent that the scavenging behaviour of “tearing” was used throughout the decomposition cycle, but most in the later stages. Whereas “biting” was observed throughout the entire decomposition process, without variation between stages.

The second group of feeding behaviours (H = 95.775) included “none” (*n* = 4 182, M = 17.91 kg), “burrowing snout” (*n* = 98, M = 38.94 kg), “twist-with-bite” (*n* = 4, M = 18.6 kg) and “licking” (*n* = 34, M = 41.46 kg). Statistically significant differences in median feeding behaviour scores were found between the pairs of “none” and “licking” (*P* = 0.002), and “none” and “burrowing snout” (*P* = 0.000). No other pairwise combinations of feeding behaviour within this group presented a statistically significant difference. Figure 3B depicts the distributions observed in this test. From these box plots, it was apparent that the “burrowing snout” behaviour was far more common in the earlier stages of decomposition; “twist-with-bite” was more often observed in the later stages of decomposition; “licking” was observed throughout all decomposition stages; and “no feeding behaviours” were observed in the later stages of decomposition.

The third group of feeding behaviours (H = 33.432) included “none” (*n* = 4 176, M = 19.28 kg), “chewing or gnawing” (*n* = 105, M = 22.49 kg), “scratching” (*n* = 37, M = 55.60 kg). The behaviours “chewing or gnawing” and “scratching” displayed a statistically significant difference in median values (*P* = 0.001) along with ‘none’ and “scratching”, which also produced a statistically significant difference (*P* = 0.000). No other pairwise combinations of feeding behaviours were significant. Figure 3C depicts the distributions observed in this test. From these box plots, it was apparent that “scratching” was most common when the porcine body weighed between 30 kg and 60 kg; “chewing or gnawing” occurred throughout the entire decomposition process; and “none” was most common in late decomposition stages.

A primary question to answer was whether specific feeding behaviours would be associated with decomposition stages. As the K-W test results confirmed, this was true for some behaviours. The “scratching” behaviour was most frequently observed during the earlier stages of decomposition. This behaviour was documented in previous research and is likely a means of creating an opening in the flesh for more robust scavenging to take place [37]. This key research finding from this facility maintains that those involved in death investigations must anticipate soft-tissue artefacts of the sort described above likely to be found early in the decomposition process. Should these artefacts be found without confirmation that the Cape grey mongoose has been in the area, the indentations caused by scratching could be mistaken for peri- or ante-mortem damage [37].

The “pulling” behaviour was consistently observed in combination with the “biting” behaviour, hence these were grouped for the K-W test. These behaviours were observed throughout the decomposition process and their evidence would likely be identifiable, provided there was still soft tissue present on the remains. The piercing of the flesh from “biting” creates punctures, followed by the “pulling” behaviour that creates a teardrop-shaped alteration. The “tearing” behaviour was defined as pulling the flesh sideways; such an action will cause a rip to form where the flesh used to be joined. This then exposed the different layers of flesh apparent on the decomposing body, as a piece would now be missing and could indicate that a scavenger has accessed the remains. Due to an absence and/or paucity of research into the lasting artefacts that different scavenging feeding behaviours leave on carrion, no comparative data are available to discuss these results.

The “twist-with-bite” behaviour was almost exclusively observed during the later stages of decomposition. The twisting component was a much faster manoeuvre than the others observed, suggesting that it required a higher amount of force. During the twist motion, soft tissue becomes bunched together and stretched until tears appear and the tissue becomes loose. This then allowed for the bite–pull combination, as the weakened structure of the tissue broke off more easily. If this behaviour has occurred at a set of remains, it may be distinguishable by a twisted bunching of soft tissue. However, over time, this bunching is likely to unfurl and resemble the typical shape of soft tissue. There may be key identifiers of this behaviour occurring in the form of small tears grouped in a circular pattern. If further scavenging does not occur to this area, these tears may remain and be identifiable.

It serves to note that this research is not (nor does it purport to be) the first to describe vertebrate scavenger behaviours, feeding preferences by tissue type or body region, or even the first to attempt to systematically describe scavenger interactions with remains. As far back as 1989, forensic taphonomists made efforts to systematically analyse scavenger effects on decomposition, with Haglund et al. [8] presenting the first description of decomposing body disarticulation sequences at the hands (or, rather, mouths) of canids in the Pacific Northwest, USA. Indra et al. [60] provide a comprehensive current review of numerous studies that have documented tissue substrate and body region preferences by vertebrate scavengers in a European context.

The above said, almost all descriptions of scavenging behaviour in the literature are qualitative (i.e., they do not *quantify* aspects of the behaviour, such as frequencies of use of specific behaviours or where on the decomposing body particular behaviours are employed). This is, however, starting to change. A good example is the work by Dibner et al. [26], who classified scavenger interactions with the decomposing body into three categories and quantified the frequencies of occurrence of each interaction category with respect to progression of decomposition (as measured by elapsed time and accumulated degree days). Another recent example is the work of Keyes et al. [61] who investigated vertebrate scavenger assemblages and behaviour in urban settings in Johannesburg, South Africa. In this study, the authors quantified the frequency with which slender mongoose (*Galerella sanguinea*) scavenged particular regions of the porcine bodies under study, and when, within a 24 h day on average, scavenging activity of this species was most prolific. Similarly, there is a growing recognition of the importance of documenting vertebrate scavenger behaviour in ever-greater detail, as clearly articulated by Smith [34]. Our work *extends and draws together* the good practices these studies represent, building upon the appreciable literature based on investigating vertebrate scavengers in forensic contexts, and emphasises the additional value that could be gained by *systematically classifying* feeding behaviours using robust definitions such that patterns of behaviours may be quantified in relation to other decompositional features (e.g., stages of decay, PMI, tissue type, body region).

### Mongooses and maggots

**Figure 4:**
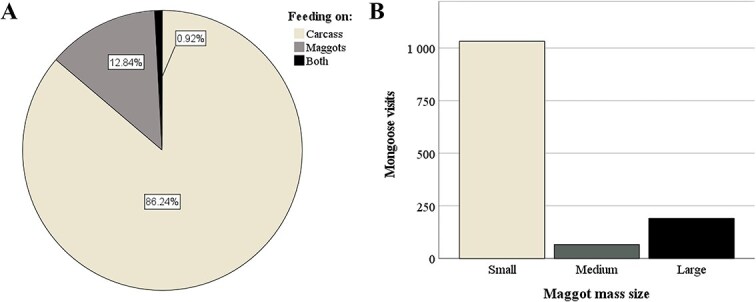
(A) A pie chart showing frequency differences between feeding on the carcass, maggots, or both. (B) An indication of the number of times mongooses visited the carcass relative to maggot mass size.

An unexpected, but novel, finding from this study was videographic confirmation that the Cape grey mongoose predates on maggot masses colonising the decomposing body. The frequencies of this activity, dependent upon maggot mass size, are depicted in Figure 4. Although feeding on maggots (*n* = 215) comprised nearly 13% of all recorded feeding events, most feeding was still devoted to body tissues (>86% of all recorded feeding events). The mongooses very rarely fed on both tissues and maggots simultaneously (<1% of all recorded feeding events). The Cape grey mongoose diet has been reported to include insects, albeit less than 10% overall [41, 62]. The findings of this research support the conclusion in the existing body of research that the Cape grey mongoose feeding regime is opportunistic, rather than systematic [40]. One drawback is that maggots were only available for a limited amount of time and the proportions of the maggot masses were ever-changing. The porcine body, by comparison, was a much more constant and stable resource, being larger and holding greater nutritional value, which may influence preference. One urban study in South Africa confirmed that carrion, as opposed to insects, eggs, and other such food items, were favoured among groups of yellow mongoose (*C. penicillata*) and slender mongoose (*G. sanguinea*) [63]. The authors also noted that aggressive, competitive behaviour was more prevalent at sites with a meat selection, suggesting that, due to its high value as a food resource requiring relatively little energy investment compared to hunting, mongooses prefer carrion over other resources.

Our study also considered whether mongoose visitation could be related to maggot mass size. Results suggested that when maggot masses were smallest, mongooses visited more frequently (Figure 4B). This observation makes sense for two reasons: firstly, maggot masses are smallest during the early stages of decomposition, when the soft-tissue is still fresher (and, therefore, more palatable for the mongooses); secondly, large maggot masses can cause the accumulation of ammonia to levels that are toxic for small mammals, potentially precipitating aversion in the mongooses [64]. It serves to note that these observations have also been made in previous research in this biogeographic region [37, 40, 44]. To the best of the authors’ knowledge, this study is the first to consider whether mongoose visitation has been influenced by key characteristics in decomposition stages, such as maggot mass size. This highlights the need for further research within this field, and for studies to collect data on multiple genera.

The confirmation that mongooses have been feeding on maggots from the decomposing body poses serious implications for PMI estimations in death investigations for the region [4, 65]. Though some studies have noted scavengers, such as raccoons (*Procyon lotor*), will feed on maggots, they have made no reference to how this implicates PMI estimation research for that locale [20, 66, 67]. The lack of international research on this topic illustrates the importance of the current study, as accurate PMI estimation is paramount in death investigations [68]. Without a classification of markers to identify the Cape grey mongoose as a scavenger of porcine bodies, there is an inability to factor into PMI estimation both scavenging and the removal of maggots from a decomposing body. Thus, the accuracy of PMI estimates can be questioned, with practitioners risking their expert submissions to court falling short of the evidentiary thresholds commonly applied in the post-Daubert era.

### Limitations, resolutions, and future research

This study was conducted with a single porcine body, thereby, limiting the scope of statistical analyses. As a pilot study intended to replicate a forensically realistic scenario and form the basis of research to come, a smaller sample was adequate for baseline outcomes. Directly, this means the research is limited to a single summer season. Validation of this research is required, employing multiple single, clothed porcine bodies deployed in replicate environments within the biogeographic region to avoid simple pseudo-replication [69]. To establish whether seasonal changes influence the feeding behaviours or body site preferences of the Cape grey mongoose, this setup should also be replicated across multiple seasons.

Lastly, due to the proximity of video cameras to the porcine body, some data may have been unintentionally omitted. Mongoose counts were only made when different mongooses could be clearly distinguished. During the bloat stage due to increased size the video frames did not always include the entire porcine body. This led to some data being indistinguishable, either because mongooses could not be observed in any video frame, or because the corresponding porcine body data were unattainable. To address this, future studies implementing similar equipment systems should include the four cameras included in this data, with the addition of a camera trap situated superiorly to the porcine body and another camera trap positioned further away, affording two independent views of the entire site. Superiorly positioned footage would assist with establishing how many mongooses are interacting with the porcine body at one time, whereas the camera further can better estimate how much time the mongooses are spending around the body.

Looking forward, the next phase of this research is to evaluate the retained porcine skeletal remains to identify any potential Cape grey mongoose-specific hard tissue artefacts. Macroscopically, the use of different light directions, such as oblique lighting, and magnification lamps may improve artefact visualisation. A powdered fluorescent dye could also be applied to exposed bone to better emphasise any surface defects that can then be visualised under ultraviolet light. Such analyses would allow us to confirm whether the identified feeding behaviours might, indeed, leave such artefacts, particularly in areas where we expect them based on the analyses presented herein. Should such artefacts be identified, prospective forensic casework of decomposed and/or skeletonised human remains in the region should include a search for these artefacts to ascertain whether they might be diagnostic of Cape grey mongoose scavenger activity on human remains. If such artefacts are found, an eDNA survey of the remains could confirm if Cape grey mongoose is the agent, towards validation of the use of hard tissue artefacts alone for the diagnosis of Cape grey mongoose scavenging of human remains in forensic cases.

## Conclusion

The volume of death investigations that Cape Town experiences on a yearly basis has created a forensic need for a greater understanding of how local scavenger guilds impact decomposition. Given that the Cape grey mongoose was identified as a scavenger of adult human-sized porcine remains deposited in forensically relevant circumstances, and that their scavenging behaviour can noticeably affect the rate and pattern of decomposition, the accuracy of PMI calculations for bodies recovered within the region may be inaccurate. Scavenging activity on remains (human and animal) has been shown to accelerate the decomposition process in diverse circumstances (not just locally), suggesting that PMI calculations may be over-estimated during death investigations where scavengers might have had access to the body. This study aimed to analyse and describe—in greater detail than previous studies—Cape grey mongoose scavenging behaviour on a single clothed porcine body in a forensically significant location in Cape Town, South Africa, to establish a methodological framework for describing and quantifying a greater level of detail in scavenger activity.

For the first time, specific feeding behaviours by this species were distinguished and described. Examining the frequency of scavenging at regions across the body has identified regions of interest that forensic anthropologists could scrutinise for signs of scavenger artefacts where a mongoose was involved (e.g., the eye sockets). We established that the decomposition state influenced the types of feeding behaviours employed. For example, scratching was often used in the earlier stages of decomposition; twist-with-bite, although not observed very often, was only employed in later stages of decomposition, suggesting that this manoeuvre was employed when the soft tissue was tougher and harder to remove. This study provided the first evidence that Cape grey mongooses will predate on porcine body-based maggot masses and that maggots appeared to affect mongoose visitation insofar as visits were most common when maggot mass size was small. The extent to which this insectivory might affect carrion entomofaunal-driven decomposition and/or entomological estimates of PMI requires further investigation.

The observations presented are noteworthy not only for regional South African forensic casework but for the international forensic taphonomic community too, as they underscore the importance of pursuing detailed study of the feeding behaviours of forensically relevant scavengers. Quantitatively accounting for scavenging in PMI estimates is impossible without studies like this one. We present methodology and equipment set-up plans to enable validation of the results and for the evaluation of scavenger behaviours amongst other forensically relevant scavenger guilds, which can also serve as a foundation for more detailed, comprehensive scavenger behaviour classification and quantification efforts. In doing so, forensic taphonomic research will move towards species-specific artefact classifications and quantitatively accounting for factors influencing the decomposition process—necessary for scientifically defensible and legally robust estimates of PMI in forensic death investigations.
